# The Association Between Financial Hardship and Mental Health Difficulties Among Adult Wage Earners During the COVID-19 Pandemic in Bangladesh: Findings From a Cross-Sectional Analysis

**DOI:** 10.3389/fpsyt.2021.635884

**Published:** 2021-09-20

**Authors:** Mst. Sadia Sultana, Abid Hasan Khan, Sahadat Hossain, Tauhidul Islam, M. Tasdik Hasan, Helal Uddin Ahmed, Zezhi Li, Jahangir A. M. Khan

**Affiliations:** ^1^Department of Public Health and Informatics, Jahangirnagar University, Dhaka, Bangladesh; ^2^Public Health Foundation, Dhaka, Bangladesh; ^3^Faculty of Veterinary, Animal and Biomedical Sciences, Sylhet Agricultural University, Sylhet, Bangladesh; ^4^Department of Primary Care and Mental Health, University of Liverpool, Liverpool, United Kingdom; ^5^National Institute of Mental Health, Sher-E-Bangla Nagar, Dhaka, Bangladesh; ^6^Department of Psychiatry, The Affiliated Brain Hospital of Guangzhou Medical University, Guangzhou, China; ^7^Health Economics and Policy Unit, School of Public Health and Community Medicine, Sahlgrenska Academy, University of Gothenburg, Gothenburg, Sweden

**Keywords:** COVID-19, anxiety, depressive symptoms, financial crisis, wage earners, Bangladesh

## Abstract

**Background:** The ongoing COVID-19 pandemic has created several challenges including financial burdens that may result in mental health conditions. This study was undertaken to gauge mental health difficulties during the COVID-19 pandemic and gain an insight into wage earners' mental health.

**Method:** This cross-sectional study was conducted through an online survey. A t total of 707 individual Bangladeshi wage earners were enrolled between 20 and 30 May 2020. The questionnaire had sections on sociodemographic information, COVID-19 related questions, PHQ-9 and GAD-7 scales. STATA version 14.1 program was used to carry out all the analyses.

**Results:** The study revealed that 58.6 and 55.9% of the respondents had moderate to severe anxiety and depressive symptoms, respectively. The total monthly income was <30,000 BDT (353.73USD) and displayed increased odds of suffering from depressive symptoms (OR = 4.12; 95% CI: 2.68–6.34) and anxiety (OR = 3.31; 95% CI: 2.17–5.03). Participants who did not receive salary income, had no income source during the pandemic, had financial problems, and inadequate food supply and were more likely to suffer from anxiety and depressive symptoms (*p* ≤ 0.01). Perceiving the upcoming financial crisis as a stressor was a potential risk factor for anxiety (OR = 1.91; 95% CI:1.32–2.77) and depressive symptoms (OR = 1.50; 95% CI:1.04–2.16).

**Limitations:** The online survey method used in this study limits the generalizability of the findings and self-reported answers might include selection and social desirability bias as a community-based survey was not possible during the pandemic.

**Conclusion:** Wage earners in a low resource setting like Bangladesh require mental health attention and financial consideration to deal with mental health difficulties.

## Introduction

The ongoing COVID-19 pandemic has resulted in many people having serious concerns about job security and financial challenges globally. With a rapidly increasing number of infected cases, the governments of most countries have declared that many public health approaches including nationwide lockdown, declaration of general holidays, and social distancing, etc. in this critical situation. However, these strategies have also been found to be the most important factors associated with death by suicide during an emerging infectious disease outbreak (1). A recent Chinese study during the COVID-19 pandemic reported that 53% of respondents rated the psychological effects of the epidemic was as moderate or severe, 16.5% had mild to extreme depressive symptoms, and 28.8% had moderate to severe anxiety symptoms (2). Another study during the pandemic among people in Indian communities revealed that people of younger age, female gender, and that comorbid physical conditions were all associated with a greater psychological effect (3). The most recent nationwide, representative survey (National Mental Health Survey 2018-19) was conducted in 2019 before the pandemic and the prevalence of any mental disorder amongst the adult population was 16.8% (4).

Two previous Bangladeshi studies before the COVID-19 pandemic showed 16.3% respondents from rural areas (5) and 28% respondents from urban areas (6) had mental health disorders whereas, economically deprived responders, those over 45 years old, and women from large families have a slightly higher incidence of psychiatric illnesses (5). It was documented before that poverty-related emotional distress can affect a person's capability to deal with economic deprivation, which contributes to psychiatric illnesses (5, 7).

More recently, few studies including some in Bangladesh and Pakistan have reported that people committed suicide for fear of COVID-19 infection, social stigma, isolation, anxiety, depression, and emotional disharmony as well as economic shutdown, financial, and future insecurities (8, 9). Therefore, it has been anticipated by the United States that the economic recession as a consequence of this ongoing pandemic is a more significant factor in terms of loss of life COVID-19 itself, as there has been a huge rise (4.6 million) in unemployment during this outbreak (10). As a result, peoples' mental health is becoming unstable. The economic fallout triggered by the pandemic needs to be researched and these potentially negative consequences examined.

While people in high-income and welfare states have access either to public funds (social benefits, unemployment benefits, sickness benefits, and universal basic income, etc.) or COVID-19 related income support from the government, the people in less developed and developing countries undoubtedly remain more vulnerable as numerous small and medium shaped businesses have been hard-hit, resulting in bankruptcy (11, 12), with these issues also affecting Bangladesh. It needs to be noted here that the governments in many low- and middle-income countries declared COVID-19 related income support, however, the situation in Bangladesh is more fragile as a report from the 2018–2019 economic year revealed that 20 and 10.5% of people in Bangladesh live below the poverty line and in extreme poverty, respectively. The unemployment rate is 4.4% among the general population and the percentage of people living from hand-to-mouth is remarkably high (70%) (13–15). This situation is likely to get more serious due to the extreme fallout in economic sectors among Bangladeshi people due to the COVID-19 emergency, as per capita income declined 82% from $1.30 (US) in February to $0.32 in early April among slum dwellers and 79% fallout from $1.05 to $0.39 among poor rural people (15). Consequently, there is a chance of a 1.10% downfall in GDP (approximately $3 billion loss in GDP), the cruel outcome of the COVID-19 pandemic (16). Additionally, the price of daily commodities is rising day by day as the production and supply have been seriously disrupted. Poor people suffer most as it is tough for them to survive with lower or no income during the pandemic situation as they experience financial uncertainty more acutely (9, 17). Price hikes and income fallout together are creating challenges for people, especially those who strive to live for the basic needs of their families. The most serious concern is that no one knows when this financial crisis, unemployment problem, as well as the concern of job insecurity, will be resolved.

There is an urgent need to understand the possible mental health issues that are faced by the wage-earning members of families as the financial challenges have risen in this outbreak. However, to the best of our knowledge, no detailed study on the mental health difficulties of earners during the pandemic has been conducted in Bangladesh. Thus, the current study seeks to explore the mental health difficulties of wage earners in the country of Bangladesh to report on anxiety and depressive symptoms and identify some potential factors linked with these mental issues during the COVID-19 outbreak.

## Methodology

### Study Design and Participants

This cross-sectional study was conducted through an online survey, a total of 707 individual Bangladeshi wage earners were enrolled between 20 May 2020 and 30 May 2020. The study was conducted following the Checklist for Reporting Results of Internet ESurvey (CHERRIES) guidelines (18). The target population of the study was Bangladeshi earners (not restricted to any division or district) living in the country during the COVID-19 outbreak. Other inclusion criteria include: willingness to participate; providing informed consent; age ≥18 years; being able to understand Bangla language. An online convenience sampling technique was chosen to meet the study aims.

### Data Collection Tool

An online semi-structured questionnaire was developed using google forms and then used as a data collection tool. The questionnaire was drafted in Bangla first. Then translation and back translation from Bangla to English and vice versa was done by bilingual experts. A pilot test was performed on 30 respondents to confirm the reliability of the questionnaire and further modifications were done in the questionnaire. Survey link was disseminated with the description of the study on ~30 Facebook groups including different alumni and organizational groups (i.e., Jahangirnagar University Alumni Association, Teacher's association), where the group members were mostly wage-earners from different occupations. An information sheet describing the aim and process, right to refuse their participation from the study was presented on the first page of the survey attaching a consent form with it. No incentive was provided to the participants for their participation in this study. The questionnaire had sections on sociodemographic information, COVID-19 related questions, and PHQ-9 and GAD-7 scales to assess depressive symptoms and anxiety, respectively. Participants were informed that their information will only be used for research purposes. Anonymity and confidentially were fully ascertained. All the participants provided written consent prior to participation. All the procedures of this study complied with the Code of Ethics of the World Medical Association (Declaration of Helsinki) for any experiments involving humans. The protocol of this study was reviewed & supported by the Department of Public Health and Informatics, Jahangirnagar University, Savar, Dhaka, Bangladesh.

### Measure

#### Socio-Demographic Measures

Socio-demographic information was gathered from all the respondents through both open-ended and closed-ended questions, including their sex, age, religion, marital status (i.e., married, unmarried, divorced, widow), educational status, occupation, monthly family income, area of residence (rural or urban), name of the district, number of family members and number of earning members in the family. For categorization of age in our study, the first category was 18–24 years taking this age group into account as youth groups (19). While the 25–35-year-old category was taken as younger adults (compared to >35 years) based on the fact that it takes ~25 years for a person to complete their educational attainment according to the Bangladeshi educational context. Lastly, people over >35 years were considered as the middle age group. The average monthly family income was categorized into three categories: (i) <30,000 BDT; (ii) 30,000–70,000BDT; (iii) >70,000BDT by considering 30,000 BDT as median income following a prior published paper from Bangladesh (20).

#### Current Income Status-Related Data

Current income status-related data were obtained through a checklist, which had 4 options such as “I am not getting any salary in this lockdown situation”; “I have no source of income currently”; “My income is not enough for my family” and “I'm satisfied with my income.” Respondents were able to choose multiple answers from this checklist.

#### Perceived Social and Financial Stressor-Related Data

Respondents were asked to choose some factors related to socio-economics that were putting them in psychological discomfort through a checklist that had options such as “I'm getting no salary in this lockdown situation,” “food supply is not enough for my family,” “dealing with the financial problem,” “future financial crisis,” “price increment in daily necessary commodities,” and “hamper my children's study.” Respondents were able to choose multiple answers from this checklist.

#### Depressive Symptoms

The nine-item scale, Bangla Patient Health Questionnaire (PHQ-9) corresponding to DSM-IV Diagnostic Criteria of symptoms for major depressive disorder was used to measure the level of depressive symptoms of the participants (21, 22). Respondents were asked to answer on a 4-point Likert scale (from “0 = not at all” to “3 = nearly every day”) based on the past 2 weeks, whereby a 0–27 score range is possible. A score of 0 indicates the absence of depressive symptoms and a total score of 27 indicates daily depressive symptoms. The five cut-off points were used for the categorization of depressive symptoms: (i) ′0–4′ for “normal”; (ii) ′5–9′ for “mild depressive symptoms”; (iii) ′10–14′ for “moderate depressive symptoms”; (iv) ′15–19′ for “moderately severe depressive symptoms”; and finally, (v) ‘20 or higher' for “severely severe depressive symptoms.” A cut-off score of ≥ 10 was set to denote “depression positive” for analysis in this study (23). In the present study, the Cronbach's alpha was 0.85.

#### Anxiety Disorder

The seven-item scale, Bangla Generalized Anxiety Disorder (GAD-7), which was reported to have good sensitivity (89%) and specificity (82%) for assessing the severity of anxiety in both the clinical and general population, was used in this study (24–26). Participants were asked how often they were bothered by the symptoms of GAD-7 based on the past 2 weeks to respond on a 4-point Likert scale the same as PHQ-9, where a score range of 0–21 was possible. The cut-off points for the categorization of the level of GAD symptoms were as follows: (i) ′0–4′ for normal, (ii) ′5–9′ for mild, (iii) ′10–14′ for moderate, and (iv) ′15–21′ for severe. A cut-off score of ≥ 10 was set to denote “anxiety positive” for analysis in this study (27). In the present study the Cronbach's alpha was high (0.84).

### Statistical Analysis

Descriptive statistics such as frequency and percentage were obtained to describe participants' characteristics. Both bivariate and multivariate logistic regression were performed to explore the potential influencing factors associated with anxiety and depressive symptoms. All variables were entered into a binary logistic regression model with “depressive symptoms” and “anxiety” as the dependent variable. The odds ratio (OR), adjusted odds ratio (28), and 95% confidence interval (95% CI) were obtained from logistic regression models. A *p* ≤ 0.05 was considered to be statistically significant for all analyses. The STATA version 14.1 program (StataCorp LP., College Station, TX, USA) was used to carry out all analyses.

## Results

### Sociodemographic Characteristics, Anxiety, and Depressive Symptoms

Responses came from 707 citizens (age range: 18–75 years, with a mean of 31.41 ± 8.73 years) from the 50 districts of Bangladesh. The majority of the respondents belonged to the age group 25–35 (62.38%), were male (77.23%), and were government or private service holders (60.82%). Most of the respondents were living in an urban setting (85.86%) in a family consisting of <5 members (43.28%) or 5–7 members (44.70%). Almost half of the respondents reported earning <30,000 BDT (48.94%) and almost one-third of the respondents were the only earner in their family (33.66%) (Table 1).

**Table 1:** Association among socio-demographics, anxiety and depressive symptoms among adult wage earners in Bangladesh.

**Variables**	***n* (*N* = 707)**	**Anxiety** ^**^§^**^		**Depressive symptoms** ^**^§§^**^	
		**OR (95% CI)**	**AOR (95% CI)**	**OR (95% CI)**	**AOR (95% CI)**
**Age (years)**					
<25	112 (15.84%)	1.26 (0.68-1.44)	1.30 (0.66–2.57)	2.29^**^ (1.38–3.83)	1.69 (0.85–3.36)
25–35	441 (62.38%)	0.99 (0.77–2.09)	1.16 (0.73–1.86)	1.19 (0.83–1.72)	1.30 (0.82–2.05)
>35	154 (21.78%)	Ref.	Ref.	Ref.	Ref.
**Sex**					
Female	161 (22.77%)	1.78^**^ (1.22–2.59)	1.69^*^ (1.11–2.58)	2.29^**^ (1.57–3.34)	1.97^**^ (1.28–3.01)
Male	546 (77.23%)	Ref.	Ref.	Ref.	Ref.
**Education**					
Up to Higher secondary	167 (23.62%)	2.30^**^ (1.57–3.37)	1.22 (0.77–1.93)	3.22^**^ (2.17–4.76)	1.95^**^ (1.22–3.10)
Honors or above	540 (76.38%)	Ref.	Ref.	Ref.	Ref.
**Occupation**					
Businessman	88 (12.45%)	3.31^**^ (1.78–6.14)	2.40^**^ (1.22–4.73)	2.66^**^ (1.47–4.81)	2.25^*^ (1.16–4.39)
Govt./Pvt. Service holders	430 (60.82%)	1.53 (0.99–2.34)	1.18 (0.74–1.88)	1.51 (0.98–2.32)	1.26 (0.79–2.01)
Others^†^	86 (12.16%)	1.46 (0.82–2.60)	1.03 (0.54–1.98)	1.99^*^(1.11–3.56)	1.41 (0.72–2.74)
HCW^‡^	103 (14.57%)	Ref.	Ref.	Ref.	Ref.
**Marital status**					
Ever married^e^	362 (51.20%)	1.36^*^(1.01–1.84)	1.32 (0.88–1.96)	0.97 (0.72–1.31)	1.00 0.67–1.48)
Unmarried	345 (48.80%)	Ref.	Ref.	Ref.	Ref.
**Living setting**					
Urban	607 (85.86%)	1.08 (0.70–1.65)	1.46 (0.91–2.34)	0.99 (0.65–1.52)	1.45 (0.90–2.33)
Rural	100 (14.14%)	Ref.	Ref.	Ref.	Ref.
**Number of family members**					
>7	85 (12.02%)	1.23 (0.75–1.99)	1.54 (0.89–2.64)	0.97 (0.60–1.58)	1.05 (0.61–1.80)
5–7	316 (44.70)	1.64^**^ (1.19–2.26)	1.76^**^ (1.23–2.51)	1.24 (0.90–1.70)	1.20 (0.84–1.71)
<5	306 (43.28%)	Ref.	Ref.	Ref.	Ref.
**Number of earning family members**					
Only earner	238 (33.66%)	1.47^*^(1.07–2.03)	1.57^*^(1.09–2.27)	1.14 (0.83–1.56)	1.25 (0.87–1.79)
More than one earner	469 (66.34%)	Ref.	Ref.	Ref.	Ref.
**Average monthly family income (BDT)**					
<30,000	346 (48.94%)	3.31^**^ (2.17–5.03)	3.12^**^ (1.90–5.17)	4.12^**^ (2.68–6.34)	2.86^**^ (1.73–4.67)
30,000–70,000	232 (32.81%)	1.67^*^(1.08–2.58)	1.78^*^(1.12–2.84)	2.11^**^ (1.35–3.30)	2.04^**^ (1.27–3.27)
>70,000	129 (18.25%)	Ref.	Ref.	Ref.	Ref.

* *P ≤ 0.05;*

** *P ≤ 0.01*.

*OR, Odds ratio; AOR, Adjusted odds ratio (adjusted for the variables only that were included in this particular table); CI, Confidence interval*.

§ *Anxiety was defined as individuals who scored ≥ 10*.

§§ *Depressive symptoms was defined as individuals who scored ≥ 10*.

† *Included researchers, journalists, farmers, tutors*.

‡ *Included doctors, nurses, and medical technologists*.

e *Included Married/divorced/widowed respondents*.

We found that 28.43% of the participants suffered from severe anxiety, while 30.13% were found to experience moderate anxiety, 24.05% were found to be experiencing mild anxiety at the time of data collection. Furthermore, it was found that 10.89% of the participants suffered from severely severe depressive symptoms, while 18.81% were found to experience moderately severe depressive symptoms, 26.17% of the participants were found to be experiencing moderate depressive symptoms, 23.48% were found to be experiencing mild depressive symptoms at the time of data collection.

Respondents aged below 25 were more likely to be experiencing depressive symptoms compared to respondents aged above 35 (OR = 2.29; 95% CI: 1.38–3.83). Females had a higher likelihood for both anxiety (AOR = 1.69; 95% CI: 1.11–2.58) and depressive symptoms (AOR = 1.97; 95% CI:1.28–3.01). Similarly, respondents earning <30,000 BDT (353.26 USD) monthly were about two or three times more likely to have depressive symptoms (AOR = 2.86; 95% CI: 1.73–4.67) and anxiety (AOR = 3.12; 95% CI: 1.90–5.17). The average monthly income ranging from 30,000 to 70,000 BDT was significantly associated with anxiety (AOR = 1.78; 95% CI: 1.12–2.84) and depressive symptoms (AOR = 2.04; 95% CI: 1.27–3.27). Apart from all these, working in business, having an education below honors and being the only earning member of the family had a significant association with anxiety or depressive symptoms or both with higher odds (Table 1).

### Current Financial Situation, Anxiety, and Depressive Symptoms

One-fourth of the respondents were not getting any salary due to the critical pandemic situation (25.32%) and they were more likely to be in anxiety (AOR = 1.69; 95% CI: 1.10–2.61) and depressive symptoms (AOR = 1.95; 95% CI: 1.27–3.00) in reference to those getting salary in some way. Similarly, respondents (34.65%) having no source of earning during the pandemic were also more likely to be in anxiety (AOR = 1.64; 95% CI: 1.11–2.43) and depressive symptoms (AOR = 2.03; 95% CI: 1.37–2.99) in reference to respondents with an earning source. Additionally, salary not being enough for family which might be reduced for the pandemic situation, and being unsatisfied with current earning had a significant association with anxiety or depressive symptoms or both with higher likelihood (Table 2).

**Table 2:** Association among current financial situation, anxiety and depressive symptoms among adult wage earners in Bangladesh.

**Variables**	***n* (*N* = 707)**	**Anxiety** ^**^§^**^		**Depressive symptoms** ^**^§§^**^	
		**OR (95% CI)**	**AOR (95% CI)**	**OR (95% CI)**	**AOR (95% CI)**
**Not getting any salary due to COVID−19**					
Yes	179 (25.32%)	2.84^**^ (1.94–4.17)	1.69^*^ (1.10–2.61)	3.05^**^ (2.09–4.45)	1.95^**^ (1.27–3.00)
No	528 (74.68%)	Ref.	Ref.	Ref.	Ref.
**No earning source**					
Yes	245 (34.65%)	2.78^**^ (1.98–3.90)	1.64^**^ (1.11–2.43)	3.09^**^ (2.21–4.32)	2.03^**^ (1.37–2.99)
No	462 (65.35%)	Ref.	Ref.	Ref.	Ref.
**Salary not enough for family**					
Yes	311 (43.99%)	2.27^**^ (1.67–3.10)	1.45 (0.97–2.16)	2.26^**^ (1.66–3.07)	1.71^**^ (1.15–2.56)
No	396 (56.01%)	Ref.	Ref.	Ref.	Ref.
**Satisfied with earning**					
No	455 (64.36%)	3.88^**^ (2.80–5.36)	2.20^**^ (1.39–3.48)	3.47^**^ (2.51–4.78)	1.58 (0.99–2.51)
Yes	252 (35.64%)	Ref.	Ref.	Ref.	Ref.

* *P ≤ 0.05;*

** *P ≤ 0.01*.

*OR, Odds ratio; AOR, Adjusted odds ratio (adjusted for the variables only that were included in this particular table); CI, Confidence interval*.

§ *Anxiety was defined as individuals who scored ≥ 10*.

§§ *Depressive symptoms was defined as individuals who scored ≥ 10*.

### Social and Financial Stressors, Anxiety, and Depressive Symptoms

About 27% of respondents reported dealing with financial problems as stressor and they had higher odds for anxiety (AOR = 2.82; 95% CI: 1.83–4.36) and depressive symptoms (AOR = 2.45; 95% CI: 1.61–3.72) in reference to their counterparts. The increased price of daily commodities (55.87%), inadequate food supply (77.93%), children's educational loss (29.42%) were significantly associated with anxiety or depressive symptoms or both with higher odds. Furthermore, respondents (79.63%) perceiving upcoming financial crisis were 2-fold more likely to be in anxiety (AOR = 2.01; 95% CI: 1.34–3.01) and 1-fold more likely to be in depressive symptoms (OR = 1.50; 95% CI: 1.04–2.16) in reference to respondents not perceiving any future financial crisis (Table 3).

**Table 3:** Association among social & financial stressors, anxiety and depressive symptoms among adult wage earners in Bangladesh.

**Variables**	***n* (*N* = 707)**	**Anxiety** ^**^§^**^		**Depressive symptoms** ^**^§§^**^	
		**OR (95% CI)**	**AOR (95% CI)**	**OR (95% CI)**	**AOR (95% CI)**
**Not getting any salary during the pandemic**					
Yes	215 (30.41%)	2.64^**^ (1.86–3.75)	2.05^**^ (1.40–3.01)	3.08^**^ (2.17–4.38)	2.45^**^ (1.68–3.57)
No	492 (69.59)	Ref.	Ref.	Ref.	Ref.
**Dealing with financial problem**					
Yes	188 (26.59%)	3.66^**^ (2.47–5.42)	2.82^**^ (1.83–4.36)	3.37^**^ (2.31–4.91)	2.45^**^ (1.61–3.72)
No	519 (73.41%)	Ref.	Ref.	Ref.	Ref.
**Increased price of daily necessary commodities**					
Yes	395 (55.87%)	1.52^**^ (1.12–2.05)	1.23 (0.87–1.72)	1.64^**^ (1.22–2.22)	1.44^*^ (1.03–2.02)
No	312 (44.13%)	Ref.	Ref.	Ref.	Ref.
**Inadequate food supply**					
Yes	551 (77.93%)	4.24^**^ (2.72–6.59)	2.27^**^ (1.39–3.70)	4.00^**^ (2.62–6.11)	2.19^**^ (1.37–3.49)
No	156 (22.07%)	Ref.	Ref.	Ref.	Ref.
**Children's educational loss**					
Yes	208 (29.42%)	1.80^**^ (1.28–2.53)	1.61^**^ (1.10–2.36)	1.65^**^ (1.18–2.31)	1.39 (0.96–2.03)
No	499 (70.58%)	Ref.	Ref.	Ref.	Ref.
**Upcoming financial crisis**					
Yes	563 (79.63%)	1.91^**^ (1.32–2.77)	2.01^**^ (1.34–3.01)	1.50^*^ (1.04–2.16)	1.47 (0.99–2.19)
No	144 (20.37%)	Ref.	Ref.	Ref.	Ref.

* *P ≤ 0.05;*

** *P ≤ 0.01*.

*OR, Odds ratio; AOR, Adjusted odds ratio (adjusted for the variables only that were included in this particular table); CI, Confidence interval*.

§ *Anxiety was defined as individuals who scored ≥ 10*.

§§ *Depressive symptoms was defined as individuals who scored ≥ 10*.

### Outcome of Multivariate Analysis With All Independent Study Variables

After having all the independent study variables in the same regression model (Table 4), some deviations from previous AORs were noticed. Through this approach, it is again noticed that females were more likely to have depressive symptoms compared to their male counterparts (AOR' = 1.67; 95% CI: 1.05–2.66), respondents in lower educational qualification category (up to higher secondary level) similarly had higher odds of having depressive symptoms than respondents with better educational qualifications (AOR' = 1.89; 95% CI: 1.15–3.12). Furthermore, respondents who were not satisfied with their earning had a significantly strong relationship with anxiety (AOR' = 2.52; 95% CI: 1.49–4.26) and depressive symptoms (AOR' = 1.72; 95% CI:1.02–2.91) in reference to respondents who were satisfied with their earning. Respondents expecting a financial crisis in the near future had their anxiety up (AOR' = 1.76; 95% CI: 1.13–2.73) but those who were dealing with the financial problems already were more likely to have anxiety (AOR' = 2.02; 95% CI:1.26–3.23) and depressive symptoms (AOR' = 1.91; 95% CI: 1.21–3.01) compared to respondents not facing or dealing with the financial problems during the pandemic. However, variables such as being the only earner of the family, having no earning source, children's educational loss alongside some other variables did not produce any significant results through this model.

**Table 4:** Adjustment of Odds-ratios (ORs) using all independent study variables.

**Variables**	***n* (*N* = 707)**	**Anxiety** ^**^§^**^		**Depressive symptoms** ^**^§§^**^	
		**OR (95% CI)**	**AOR (95% CI)**	**OR (95% CI)**	**AOR (95% CI)**
**Socio-demographics**					
**Age (years)**					
<25	112 (15.84%)	1.26 (0.68–1.44)	1.18 (0.57–2.47)	2.29^**^ (1.38–3.83)	1.58 (0.76–3.27)
25–35	441 (62.38%)	0.99 (0.77–2.09)	1.23 (0.74–2.06)	1.19 (0.83–1.72)	1.41 (0.85–2.33)
>35	154 (21.78%)	Ref.	Ref.	Ref.	Ref.
**Sex**					
Female	161 (22.77%)	1.78^**^ (1.22–2.59)	1.41 (0.89–2.23)	2.29^**^ (1.57–−3.34)	1.67^*^ (1.05–2.66)
Male	546 (77.23%)	Ref.	Ref.	Ref.	Ref.
**Education**					
Up to Higher secondary	167 (23.62%)	2.30^**^ (1.57–3.37)	1.14 (0.69–1.89)	3.22^**^ (2.17–4.76)	1.89^**^ (1.15–3.12)
Honors or above	540 (76.38%)	Ref.	Ref.	Ref.	Ref.
**Occupation**					
Businessman	88 (12.45%)	3.31^**^ (1.78–6.14)	1.38 (0.66–2.90)	2.66^**^ (1.47–4.81)	1.35 (0.65–2.78)
Govt./Pvt. Service holders	430 (60.82%)	1.53 (0.99–2.34)	1.00 (0.61–1.64)	1.51 (0.98–2.32)	1.08 (0.66–1.78)
Others^†^	86 (12.16%)	1.46 (0.82–2.60)	0.77 (0.37–1.57)	1.99^*^(1.11–3.56)	1.11 (0.54–2.29)
HCW^‡^	103 (14.57%)	Ref.	Ref.	Ref.	Ref.
**Marital status**					
Ever married^e^	362 (51.20%)	1.36^*^(1.01–1.84)	1.15 (0.74–1.78)	0.97 (0.72–1.31)	0.85 (0.55–1.32)
Unmarried	345 (48.80%)	Ref.	Ref.	Ref.	Ref.
**Living setting**					
Urban	607 (85.86%)	1.08 (0.70–1.65)	1.58 (0.96–2.63)	0.99 (0.65–1.52)	1.60 (0.96–2.65)
Rural	100 (14.14%)	Ref.	Ref.	Ref.	Ref.
**Number of family members**					
>7	85 (12.02%)	1.23 (0.75–1.99)	1.10 (0.60–2.02)	0.97 (0.60–1.58)	0.72 (0.40–1.32)
5–7	316 (44.70)	1.64^**^ (1.19–2.26)	1.57^*^(1.07–2.30)	1.24 (0.90–1.70)	1.02 (0.70–1.49)
<5	306 (43.28%)	Ref.	Ref.	Ref.	Ref.
**Number of earning family members**					
Only earner	238 (33.66%)	1.47^*^(1.07–2.03)	1.41 (0.94–2.10)	1.14 (0.83–1.56)	1.06 (0.72–1.58)
More than one earner	469 (66.34%)	Ref.	Ref.	Ref.	Ref.
**Average monthly family income (BDT)**					
<30,000	346 (48.94%)	3.31^**^ (2.17–5.03)	1.63 (0.94–2.83)	4.12^**^ (2.68–6.34)	1.48 (0.85–2.56)
30,000–70,000	232 (32.81%)	1.67^*^(1.08–2.58)	1.37 (0.83–2.26)	2.11^**^ (1.35–3.30)	1.64 (0.99–2.72)
>70,000	129 (18.25%)	Ref.	Ref.	Ref.	Ref.
**Current (during COVID−19 pandemic) financial status**					
**Not getting any salary due to COVID−19**					
Yes	179 (25.32%)	2.84^**^ (1.94–4.17)	1.29 (0.70–2.35)	3.05^**^ (2.09–4.45)	1.20 (0.67–2.16)
No	528 (74.68%)	Ref.	Ref.	Ref.	Ref.
**No earning source**					
Yes	245 (34.65%)	2.78^**^ (1.98–3.90)	1.05 (0.64–1.72)	3.09^**^ (2.21–4.32)	1.32 (0.81–2.15)
No	462 (65.35%)	Ref.	Ref.	Ref.	Ref.
**Salary not enough for family**					
Yes	311 (43.99%)	2.27^**^ (1.67–3.10)	0.81 (0.50–1.29)	2.26^**^ (1.66–3.07)	1.12 (0.71–1.78)
No	396 (56.01%)	Ref.	Ref.	Ref.	Ref.
**Satisfied with earning**					
No	455 (64.36%)	3.88^**^ (2.80–5.36)	2.52^**^ (1.49–4.26)	3.47^**^ (2.51–4.78)	1.72^*^(1.02–2.91)
Yes	252 (35.64%)	Ref.	Ref.	Ref.	Ref.
**Financial and social stressors**					
**Not getting any salary during the pandemic**					
Yes	215 (30.41%)	2.64^**^ (1.86–3.75)	1.17 (0.65–2.12)	3.08^**^ (2.17–4.38)	1.38 (0.78–2.44)
No	492 (69.59)	Ref.	Ref.	Ref.	Ref.
**Dealing with financial problem**					
Yes	188 (26.59%)	3.66^**^ (2.47–5.42)	2.02^**^ (1.26–3.23)	3.37^**^ (2.31–4.91)	1.91^**^ (1.21–3.01)
No	519 (73.41%)	Ref.	Ref.	Ref.	Ref.
**Increased price of daily necessary commodities**					
Yes	395 (55.87%)	1.52^**^ (1.12–2.05)	1.29 (0.89–1.86)	1.64^**^ (1.22–2.22)	1.30 (0.90–1.88)
No	312 (44.13%)	Ref.	Ref.	Ref.	Ref.
**Inadequate food supply**					
Yes	551 (77.93%)	4.24^**^ (2.72–6.59)	1.85^*^(1.08–3.70)	4.00^**^ (2.62–6.11)	1.67^*^(1.01–2.79)
No	156 (22.07%)	Ref.	Ref.	Ref.	Ref.
**Children's educational loss**					
Yes	208 (29.42%)	1.80^**^ (1.28–2.53)	1.55 (0.97–2.49)	1.65^**^ (1.18–2.31)	1.54 (0.97–2.47)
No	499 (70.58%)	Ref.	Ref.	Ref.	Ref.
**Upcoming financial crisis**					
Yes	563 (79.63%)	1.91^**^ (1.32–2.77)	1.76^**^ (1.13–2.73)	1.50^*^1.04–2.16)	1.31 (0.84–2.02)
No	144 (20.37%)	Ref.	Ref.	Ref.	Ref.

* *P ≤ 0.05;*

** *P ≤ 0.01*.

*OR, Odds ratio; AOR, Adjusted odds ratio; AOR', Adjusted odds ratio (adjusted for all independent variables); CI, Confidence interval*.

§ *Anxiety was defined as individuals who scored ≥ 10*.

§§ *Depressive symptoms was defined as individuals who scored ≥ 10*.

† *Included researchers, journalists, farmers, tutors*.

‡ *Included doctors, nurses, and medical technologists*.

e *Included Married/divorced/widowed respondents*.

## Discussion

This study upholds the most updated scenario of Bangladeshi wage-earners' mental health difficulties amid a period of financial stress during the ongoing COVID-19 pandemic. The findings of the present study indicate that anxiety and depressive symptoms are high among wage earners, with a prevalence of 58.6 and 55.9%, respectively. There was a clear upward trend in the prevalence of anxiety and depressive symptoms compared to previous studies conducted during the COVID-19 outbreak (29, 30). A study on Bangladeshi job seekers found a greater percentage of depression (81.1%) and anxiety (61.5%) compared to the present study (31). Two prior studies on Bangladeshi peoples' mental health during COVID-19 revealed comparatively lower depression (33%) (32) and anxiety (37.3%) (33) compared to the present study. On the contrary, another study on Bangladeshi adult peoples' mental health during the pandemic showed a higher percentage of depression (57.9%) than the present study (34).

A study which was conducted on workers in United State found that 16% of the respondents had depression (35), 26.3% depression was found in a population-based study in German (36), 0.4–15.7% depression was found in a multilevel study of 187,496 individuals from 53 countries (37). All of the aforementioned studies reported less prevalence of depression compared to the present study. The financial crisis and socioeconomic stressors may play a significant role behind the high percentage of anxiety and depression among respondents. Previously increased depression was reported in Greece due to economic crisis (38, 39), all of the prevalences of depression were lower than the present study. This elevated anxiety and depression prevalence compared to previous studies might be attributed to different socio-economic background and distinctions in the health care system. Moreover, being wage earner might be a cruicial factor acting behind the higher anxiety and depression prevalences in the present study as they meet all financial responsibilities in the family even in this critical pandemic situation where joblessness and insufficient income are predominant.

This study identified eight critical risk factors behind earners' anxiety and depressive symptoms such as “being female,” “being in younger age (<25 years),” “having lower education,” ‘working in business,” “belonging to the middle-sized family (5–7 members),” “being the only earner of the family,” “family income both <30,000BDT (353.73USD) and ranging from 30,000BDT to 70,000BDT compared to >70,000BDT.” The present study reported a much higher OR depressive symptoms and anxiety among younger earners (<25 years) than the older ones, which is identical to previously published studies reporting that psychological symptoms are linked to younger age (40). The economic crisis might appear as less depressing to older adults due to their greater maturity, patience, and experience derived from the vicissitudes of life (40). Furthermore, elevated rates (11.92%) of youth unemployment in Bangladesh can make younger adults more prone to depression compared to older individuals (41).

In agreement with the findings of previous reports and studies (29, 42), the present study found that female earners were 1.78 times more likely to be anxious and 2.29 times more likely to be depressed than the male counterparts. Working females are responsible for dealing with both household responsibilities and work responsibilities in Bangladesh, meaning they are burdened with different stresses, which may be the reason for increased anxiety and depressive symptoms. Further research is required to clarify the reasons and mechanisms behind this finding. For example, it has been suggested that women might be sensitive to stress hormones, which could affect their reaction to stressful situations (43).

The findings of our study agree with the observation that higher education works as a protective factor for both anxiety and depression (44). A higher level of education can buffer negative psychological effects, as it provides cognitive abilities to cope, and is often a conducive and less precarious social position, which decreases the negative impact of a stressful life situation on the psychological state (45). Furthermore, cognitive skills, attitudes, and shared values regarding health-related behaviors are insights from education that buffers against depression (46).

Both unadjusted and adjusted estimates in regression analysis show that earners who worked in business had higher odds of both anxiety and depressive symptoms. Some job holders were still getting a salary, but people who work in the business industry often had limited sources of income in the pandemic situation and are going through a drastic loss. A previous report claimed that worrying about cash flow is mentally challenging for business owners (47). As consistently reported previously, the present study reveals that respondents who were married had a more significant link with anxiety (48). According to the Family Stress Model, spouses' distress increases when dealing with daily economic difficulties, resulting in negative feelings like unhappiness, grudges, and disappointment about the future, which could be the reason for marital conflicts and less supportive behavior for partners. Marital conflict has therefore been reported as being linked to mental health problems (49). The study found that in medium-size families with 5–7 family members the risk was twice as high as in the smaller size families. However, the risk of having anxiety symptoms is the same in families smaller than five as those with more than seven family members. This finding might be attributable to the fact that bigger families (of >7 members) are likely to have more secure financial positions with more active earners, compared to medium-sized families, which plays a significant role in lessening burdens on mental health. Our study revealed a significant finding that respondents who were the only earning member in the entire family were 1.47 times more likely to be anxious.

Our results are in line with the results of other investigations (44, 50, 51), which reported that those who had a comparatively low salary (<30,000BDT in this study) had a higher likelihood of being depressed and anxious. A study reported that low-income elderly were 2.35 times more likely to experience depressive symptoms compared to their higher-income counterparts, which is somewhat identical to the present study (52). Again, respondents whose salary was not enough for their family had significantly higher ORs for anxiety and depressive symptoms. With regards to depressive symptoms, income was more strongly associated with depressive symptoms than education, which was observed in another previous study (52). The reason for the lower prevalence of depressive symptoms and anxiety may be that higher-income jobs allow for greater social prestige and the working conditions tend to be better from both psychological and physical perspectives (53).

In terms of current income status, consistent with our expectation, losing income almost triples the probability of mental symptoms while being dissatisfied with income almost quadruples it. However, a recent Bangladeshi study showed that the economy and jobs were significantly affected by the perception that the pandemic disrupted life events (34). It also suggests that respondents who had no source of income were more likely to be anxious and depressed. These results may be relevant for earners in other developing countries, as lockdown due to the COVID-19 pandemic has significantly disrupted employment status. Other studies were in agreement with this finding, reporting that unemployment had a negative impact on mental health (40, 54). Another epidemiological study found that unemployed people had an elevated prevalence of major depression at 58 and 53% compared to economically active and inactive people, respectively (38). This study also showed that respondents who were not satisfied with their income were suffering from anxiety and depressive symptoms. This could be because of concern about the future financial crisis, as 79.63% of respondents perceived the upcoming financial crisis as a stressor. Financial uncertainty was reported as significantly associated with anxiety and depression (17). The current study explored the association between financial hardship and mental health difficulties. Results indicated that the “dealing with financial problems” variable was very significantly associated with anxiety and depressive symptoms. A previous study mentioned that individuals displayed increased odds of suffering from major depression in 2011 when they experienced financial distress (39).

Our findings also indicate a relatively strong association between inadequate food supply and anxiety and depressive symptoms. In agreement with the findings of the present study, Butterworth et al. reported in 2009 that food insecurity causes the most serious mental health effects (50). Furthermore, perceiving inadequate food supply as a stressor was significantly associated with both anxiety and depressive symptoms, which is consistent with a recent study which was conducted on Bangladeshi students (17). Although the multivariate analysis did not find that any significant relationship of the stressor “increased price in daily commodities” with anxiety, it was found to be strongly associated with depressive symptoms even in multivariate regression. We are unaware of any previous studies in Bangladesh evaluating anxiety and depression in pandemic situations among wage earners, and are unable to compare our results with previous data from Bangladesh. Respondents who reported their children's educational loss as a stressor were found to be depressed and anxious. This finding will hopefully help in making suitable policies with significant relevance. Finally, it is worth mentioning that the upcoming financial crisis, which was also a stressor for earners, was found to be associated with both anxiety and depressive symptoms. This major finding has implications for dealing with future mental health crises, both during and after this ongoing pandemic, as the effects of financial loss are not temporary.

### Strength and Limitations

The study reported on the pandemic-related economic crisis, examining the mental state of the earning members of families. This subject has received little research attention, particularly in the context of Bangladesh. To our best knowledge, no other study has been published on this issue with this specific population.

The strength of this study lies in the use of validated assessment tools to measure mental health difficulties. However, the study has several limitations that should be taken into account. The major limitation of this study is that participation in the study required participants to have access to smartphones or a computer, which indicates that the respondents from lower socio-economic subgroups could not be included. Considering the online data collection methods and smaller sample sizes, our study is not nationally representative. Secondly, this study relies on self-reported data and was not free of recall or report biases. Thirdly, the possibility of selection bias should be considered, as the study was conducted online using a convenience sampling technique. Fourthly, the study did not assess whether the respondents suffered from COVID-19 or they had any mental health conditions prior to the pandemic. Finally, the cross-sectional design of the study includes method bias as a causal relationship, which cannot be elucidated accurately in this design. Future qualitative and longitudinal studies are needed to uncover scenario in the context of the COVID-19 pandemic.

## Conclusion

The study provides important findings that wage earners may experience an elevated level of anxiety and depressive symptoms during the COVID-19 outbreak. Current income status, socio-economic stressors & negative perception of the earners about the emerging financial stress played a significant role to develop mental health difficulties. This upward trend of anxiety and depressive symptoms indicates that wage earners in society require adequate mental health support. Furthermore, financial consideration from the state or their workplace may help them to deal with mental health difficulties during this unprecedented crisis. The findings of our study will help the frontline practitioners, mental health professionals, and policymakers to deal with the negative costs of the pandemic on the wage earners' mental health.

## Data Availability Statement

The raw data supporting the conclusions of this article will be made available by the authors, without undue reservation.

## Ethics Statement

The studies involving human participants were reviewed and approved by the Ethical Board of Jahangirnagar University, Savar, Dhaka, Bangladesh. The patients/participants provided their written informed consent to participate in this study.

## Author Contributions

MS: conceptualization, methodology, investigation, data curation, writing—original draft, writing—review and editing, and validation. AK: investigation, formal analysis, data curation, writing—original draft, and validation. SH: conceptualization, supervision, writing—review and editing, and validation. TI: investigation, data curation, writing—original draft, and validation. MH, HA, JK, and ZL: writing—review and editing and validation. All authors contributed to the article and approved the submitted version.

## Conflict of Interest

The authors declare that the research was conducted in the absence of any commercial or financial relationships that could be construed as a potential conflict of interest.

## Publisher's Note

All claims expressed in this article are solely those of the authors and do not necessarily represent those of their affiliated organizations, or those of the publisher, the editors and the reviewers. Any product that may be evaluated in this article, or claim that may be made by its manufacturer, is not guaranteed or endorsed by the publisher.
